# Role of small proliferative adipocytes: possible beige cell progenitors

**DOI:** 10.1530/JOE-19-0503

**Published:** 2020-01-28

**Authors:** Koichiro Taguchi, Kazuo Kajita, Yoshihiko Kitada, Masayuki Fuwa, Motochika Asano, Takahide Ikeda, Toshiko Kajita, Tatsuo Ishizaka, Itaru Kojima, Hiroyuki Morita

**Affiliations:** 1 Department of General Internal Medicine, Gifu University Graduate School of Medicine, Gifu, Japan; 2 Department of General Internal Medicine and Rheumatology, Gifu Municipal Hospital, Gifu, Japan; 3 Laboratory of Cell Physiology, Institute for Molecular and Cellular Regulation, Gunma University, Gunma, Japan

**Keywords:** adipocyte differentiation, adipose progenitor cells, proliferin, beige cells

## Abstract

Despite extensive investigation, the mechanisms underlying adipogenesis are not fully understood. We previously identified proliferative cells in adipose tissue expressing adipocyte-specific genes, which were named small proliferative adipocytes (SPA). In this study, we investigated the characteristics and roles of SPA in adipose tissue. Epididymal and inguinal fat was digested by collagenase, and then SPA were separated by centrifugation from stromal vascular cells (SVC) and mature white adipocytes. To clarify the feature of gene expression in SPA, microarray and real-time PCR were performed. The expression of adipocyte-specific genes and several neuronal genes was increased in the order of SVC < SPA < mature white adipocytes. In addition, proliferin was detected only in SPA. SPA differentiated more effectively into lipid-laden cells than SVC. Moreover, differentiated SPA expressed uncoupling protein 1 and mitochondria-related genes more than differentiated SVC. Treatment of SPA with pioglitazone and CL316243, a specific β3-adrenergic receptor agonist, differentiated SPA into beige-like cells. Therefore, SPA are able to differentiate into beige cells. SPA isolated from epididymal fat (epididymal SPA), but not SPA from inguinal fat (inguinal SPA), expressed a marker of visceral adipocyte precursor, WT1. However, no significant differences were detected in the expression levels of adipocyte-specific genes or neuronal genes between epididymal and inguinal SPA. The ability to differentiate into lipid-laden cells in epididymal SPA was a little superior to that in inguinal SPA, whereas the ability to differentiate into beige-like cells was greater in inguinal SPA than epididymal SPA. In conclusion, SPA may be progenitors of beige cells.

## Introduction

Numerous investigations have contributed to the elucidation of adipocyte differentiation. It has been proposed that adipogenesis, which comprises the whole process of adipocyte formation, consists of at least two steps, determination of adipose progenitor cells (APC) as the initiation of differentiation and emergence of adipose-specific genes as the terminal differentiation.

Studies using fluorescence-activated cell sorting and lineage tracing have characterized APC in white adipose tissue. A recent study revealed functional heterogeneity of stromal vascular cells (SVC). Hepler *et al.* demonstrated that LY6C^-^, CD9^-^, and PDGFRβ^+^ cells represented a highly adipogenic adipocyte precursor, whereas LY6C^+^ and PDGFRβ^+^ cells represented fibro-inflammatory phenotypes (Hepler *et al.* 2018). On the other hand, Wilms tumor gene, *Wt1*, was expressed specifically in visceral adipocyte progenitors (Chau *et al.* 2014). These results indicate that the progenitors of visceral adipocytes differ from those of s.c. adipocytes.

Recent studies have demonstrated that cold exposure or chronic stimulation with β-adrenergic agonist leads to the appearance of morphologically and functionally brown adipocyte-like cells, namely beige cells, in white adipose tissue (Vitali *et al*. 2012, Chen *et al.* 2013). Uncoupling protein 1 (UCP1) is a major marker of beige cells, as well as brown adipose tissue (BAT). Despite numerous similarities, however, BAT and beige cells differ in several aspects (Harms & Seale 2013). A lineage tracing study revealed that *Myf5*, previously thought to be expressed in myocyte precursor, was also expressed in BAT precursor. On the other hand, the origin and development of beige cells are more complicated. A study using AdipoChaser mice demonstrated that cold exposure-induced beige cells were derived from progenitor cells in s.c. fat (Wang *et al*. 2013). In addition, research to explore the progenitor of beige cells has demonstrated that precursors of beige cells express *Cd137* and *Tbx1*, but not *Myf5* and* Zic1*, markers of classical BAT (Seal *et al.* 2008, de Jong *et al.* 2015), documenting that they are distinct from BAT. A lineage tracing study revealed that PDGFRα^+^ cells differentiate into both white adipocytes and beige cells in abdominal fat (Lee *et al*. 2012). Numerous investigations have shown that the activity of BAT and beige cells is inversely correlated with obesity in mice (Vargas-Castillo *et al*. 2017), and so the applicability of this fact to humans as well has been expected (Cypess *et al*. 2015). Understanding these thermogenic adipocytes is a key to developing novel treatments for obesity.

On the other hand, while studying cell proliferation of adipose tissue, we found small round cells displaying proliferative activity and expressing adipocyte-specific genes such as adiponectin and leptin in dispersed adipose tissue (Hanamoto *et al.* 2013, Kajita *et al*. 2013). These cells do not fit into the conventional schema of adipocyte differentiation, which prompted us to name such cells expressing adipocyte-specific genes and proliferative capacity as ‘small proliferative adipocytes’ (SPA). We previously found several pieces of evidence confirming that these cells do exist, and we speculated that SPA are intermediate cells in the process of differentiation to adipocytes from stem cells. However, the characteristics and functions of these cells were not clarified, since we were unable to isolate SPA. Subsequently, we developed a new method to isolate SPA and in this way the existence of SPA became more certain. In this study, we investigated for the first time the characteristics of SPA in terms of their gene expression and ability to differentiate into adipocytes to establish their roles in adipogenesis.

## Materials and methods

### Preparation of SPA

Full details of the preparation of SPA are given in the Supplementary Materials and methods (see section on supplementary materials given at the end of this article). In brief, dispersed adipose tissue obtained by collagenase digestion was centrifuged at 8 ***g*** for 1 sec. The floating cell fraction was further centrifuged at 226 ***g*** for 3 min. The resulting floating cells and sedimentary cells were regarded as fraction A and fraction B, respectively. Cells in the lower phase of the 8 ***g*** centrifugation were regarded as fraction C.

Animal care and experimental procedures were performed with the approval of the Animal Care Committee of Gifu University Graduate School of Medicine.

### Immuno-histochemical and immuno-cytochemical study

Full details of the immuno-histochemical and immuno-cytochemical studies are given in the Supplementary Materials and methods. In brief, for immuno-histochemical studies paraffin-embedded sections were deparaffinized and antigen retrieval was performed. For immuno-cytochemical studies, 5000 cells/cm^2^ were plated on gelatin-coated glass bottom plates, and cultured. The cells were fixed and permeabilized. The samples were sequentially incubated with primary antibody, secondary antibody and DAPI. They were observed with a confocal laser scanning microscope (LSM710; Carl Zeiss).

### Quantitative PCR

Samples for Quantitative PCR were obtained from adipose tissue and cultured cells. Cells in fraction B and C obtained from 20 mice were plated in 6-well plates and cultured with DMEM until they reached 90% of confluence. Quantitative PCR was performed as described in the Supplementary material. Oligonucleotide primers are shown in Supplementary Table 1.

### Immunoblot analysis

Samples for immunoblot were obtained from adipose tissue and cultured cells. Cells in fraction B and C obtained from 10 mice were plated in flask (25 cm^2^) and cultured with DMEM until they reached 90% confluence. Cell lysate in one flask was referred to one sample for immunoblot analysis. Three samples were obtained from independent cell cultures. Immunoblot analysis was performed as described in the Supplementary material.

### Microarrays

Total RNA was purified as described. Microarrays were performed with a Low Input Quick Amp Labeling kit (Agilent Technologies, SurePrint G3 Mouse 8 × 60K ver. 2.0 (Agilent Technologies), and scanned with an Agilent DNA Microarray Scanner (G2505C). Resultant data were analyzed with GeneSpring GX (Ver.12.6).

### Flow cytometry

For flow cytometry (FCM) analysis, cells in fraction D (SVC + SPA consisting of SVC and SPA) were suspended in PBS containing 1% FBS. Cells were incubated with or without (negative control) mouse anti-AQP7 antibody (Santa Cruz) and rabbit anti-ADRB3 antibody (Abcam) antibodies listed in Supplementary Table 2 for 30 min. They were washed twice with PBS, followed by incubation with secondary antibody for 30 min at room temperature. FCM was performed with EC800 Cell analyzer (SONY, Tokyo, Japan).

### Statistics

**Figure 1:**
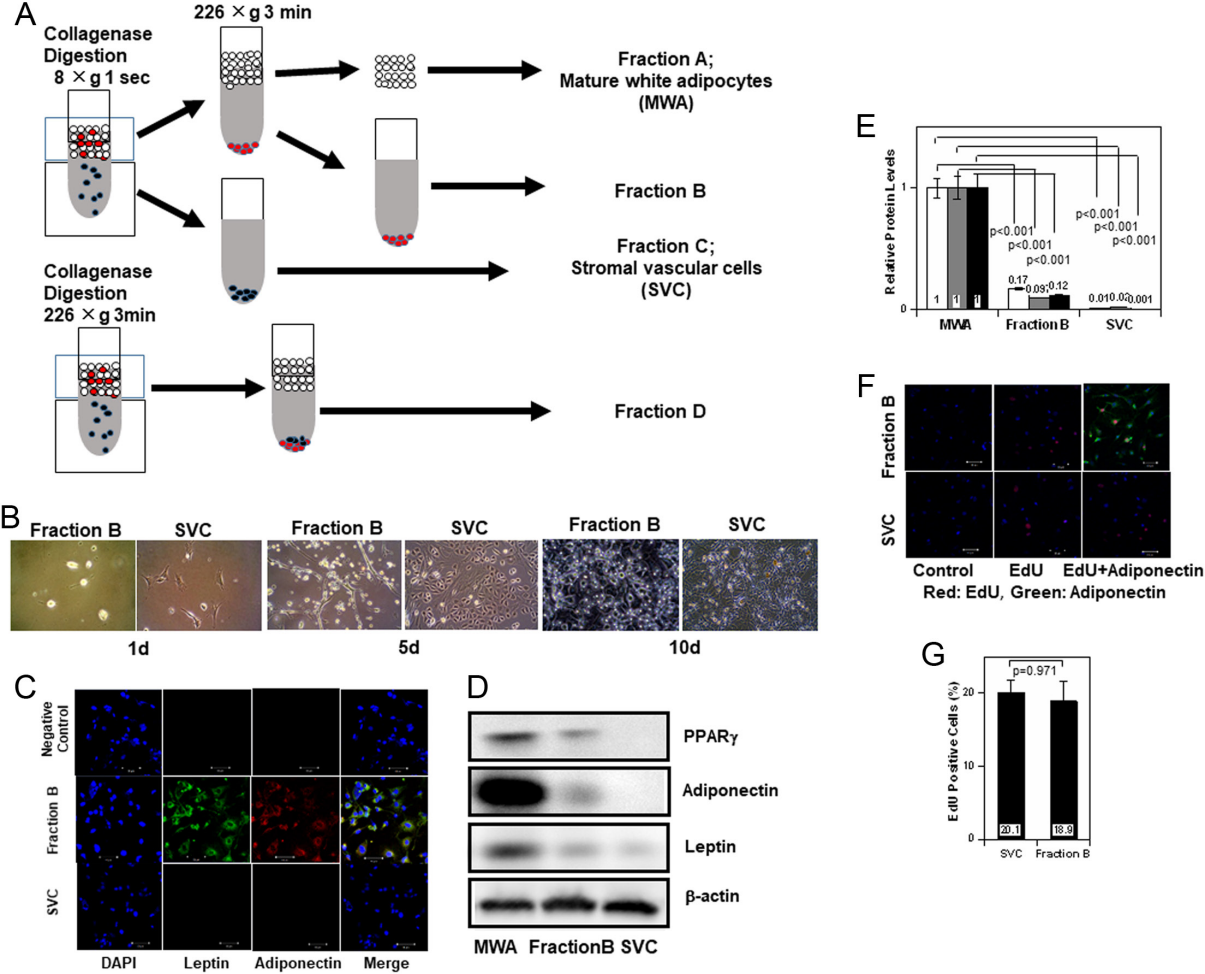
Preparation of SPA. (A) Method for preparation of SPA rich fraction (fraction B), stromal vascular cells (SVC), and mature white adipocytes (MWA). The fraction combined fraction B and C was regarded as fraction D. (B) Morphology of fraction B and SVC cultured for 1 day (left), 5 days (middle), and 10 days (right). (C) Expression of leptin and adiponectin in cultured fraction B and SVC. (D and E) Protein levels of PPARγ, adiponectin, and leptin in SVC, fraction B, and MWA were evaluated by immunoblot analysis. Typical immunoblots (D) and quantified results (E) are shown. Each value shows the average of the relative protein levels (PPARγ white, adiponectin: gray, leptin: black) (*n* = 3) (MWA as 1). (F) Incorporation of EdU into cells in fraction B and SVC. Control: EdU was not added to the medium before visualization. EdU: EdU was added to the medium before visualization. EdU + Adiponectin: EdU was added to the medium before visualization and subsequent immunostaining was performed with anti-adiponectin antibody. Red: EdU, Green: adiponectin. Scale: 50 µm (G) Percentage of EdU-positive cells/total cells in SVC and fraction B (*n* = 3).

**Figure 2:**
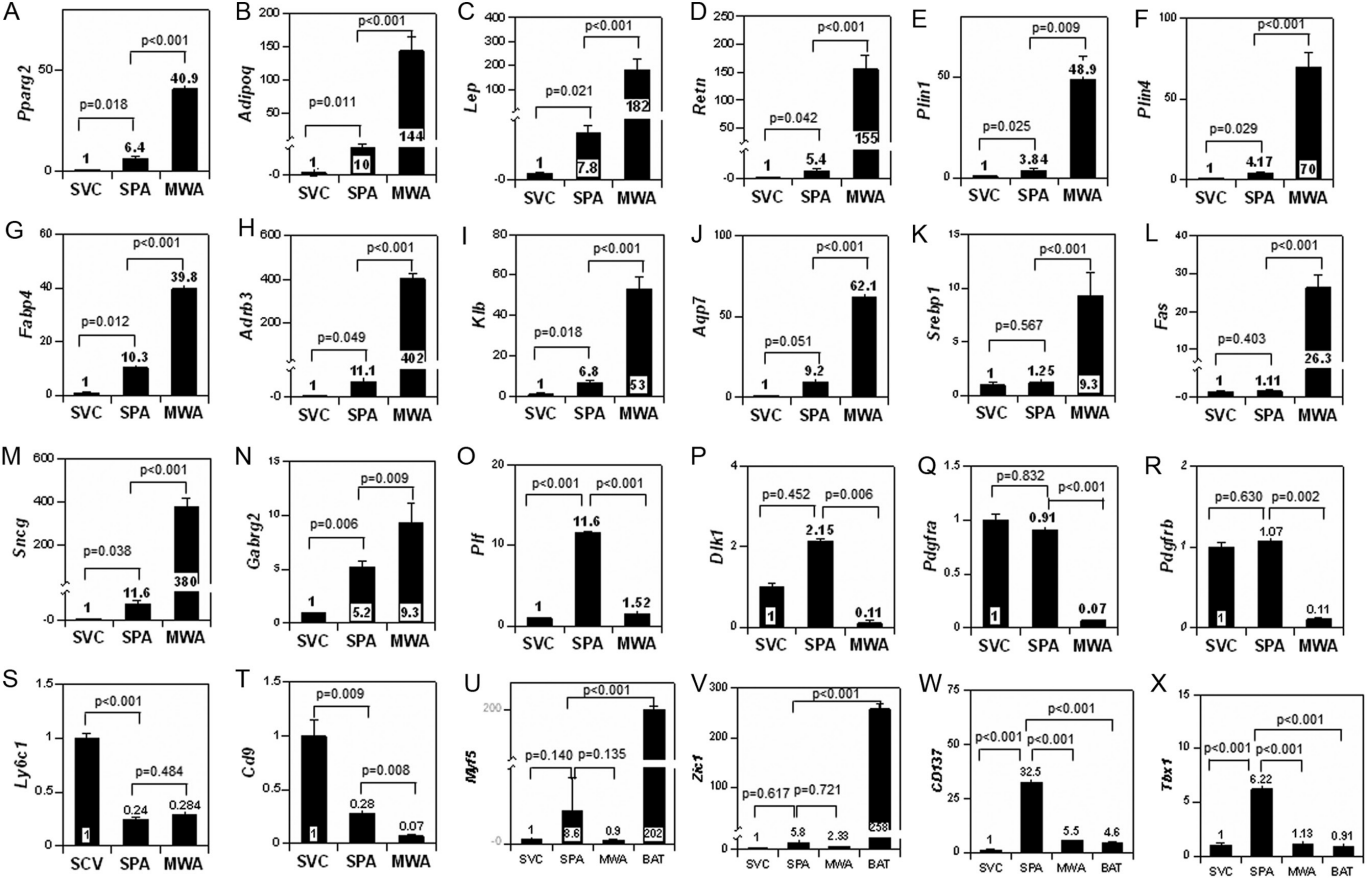
Gene expression of SVC, SPA, and MWA. Results of real-time PCR are demonstrated. (A)* Pparg2*, (B)* Adipoq*, (C) *Lep*, (D) *Retn*, (E) *Plin1*, (F) *Plin4*, (G) *Fabp4*, (H) *Adrb3*, (I) *Klb*, (J) *Aqp7*, (K)* Srebp1*, (L) *Fas*, (M) *Sncg*, (N)* Gabrg2*, (O) *Plf*, (P)* Dlk1*, (Q)* Pdgfra*, (R) *Pdgfrb*, (S) *Ly6c1*, (T) *Cd9*, (U) *Myf5*, (V) *Zic1*, (W), *Cd137*, and (X) *Tbx1*. All values represent mean ± s.e. (*n* = 5) of relative expression levels of mRNA (SVC as 1). Relative mRNA levels in brown adipose tissue (BAT) were added in U–X.

**Figure 3:**
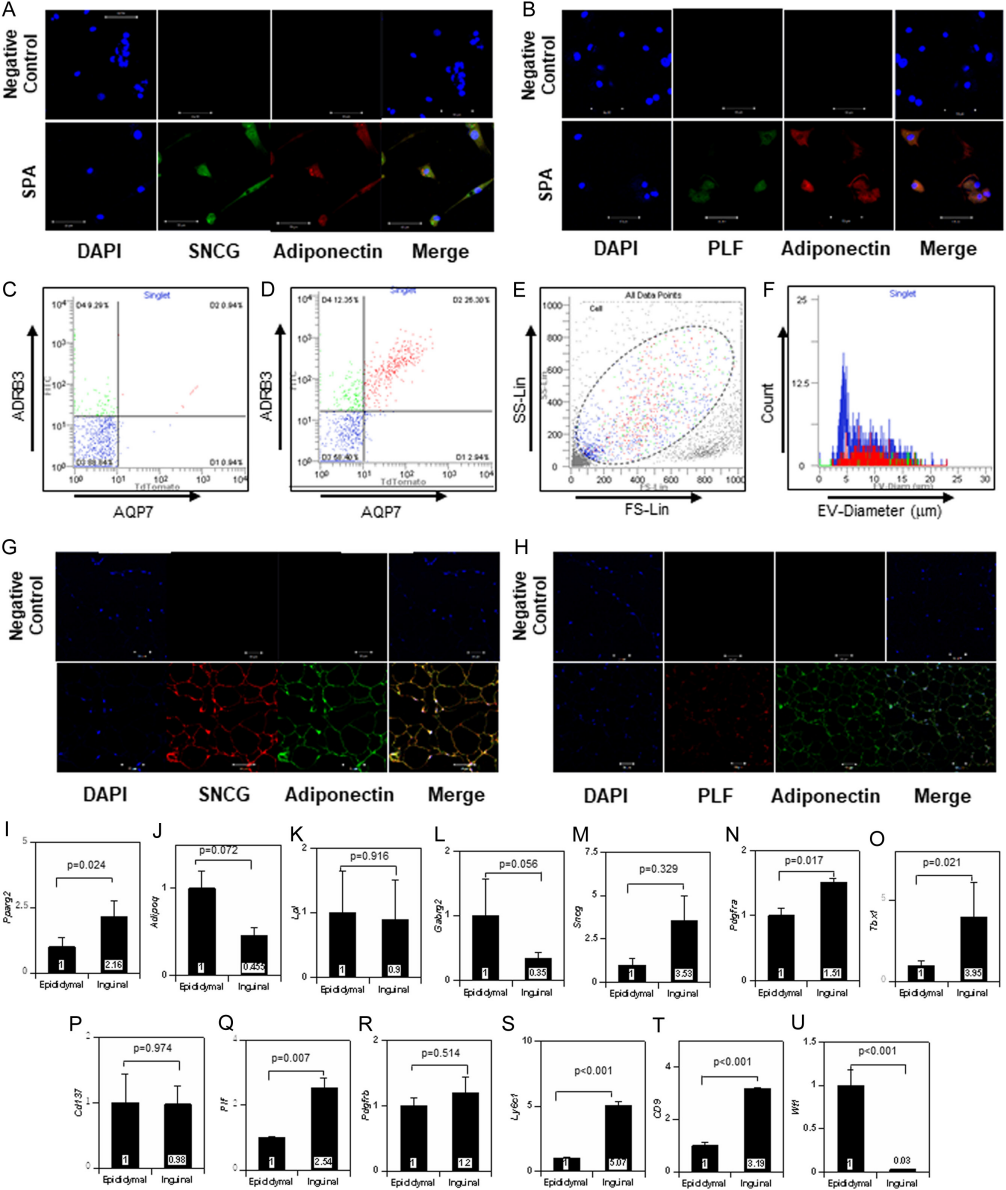
Expression of neuronal proteins and proliferin in SPA and adipose tissue. (A) Expression of SNCG and adiponectin in SPA. (B) Expression of PLF and adiponectin in SPA. (C, D, E, and F) Flow cytogram of AQP7^+^, ADRB3^+^ cells in fraction D isolated from epididymal adipose tissue. (C) Negative control, cells were incubated with the secondary antibodies. (D) Representative result cells were incubated with anti-AQP7 antibody and anti-ADRB3 antibody followed by incubated with secondary antibody. AQP7^+^, ADRB3^+^: red spots, AQP7^+^, ADRB3^+^: green spots (E) Distribution of AQP7^+^, ADRB3^+^ cells, and AQP^-^, ADRB3^+^ cells in SS/LS plot (F) Distribution of AQP7^+^, ADRB3^+^ cells and AQP^-^, ADRB3^+^ cells in cell diameter (G) Expression of SNCG and adiponectin in epididymal fat. (H) Expression of PLF and adiponectin in epididymal fat. Scale: 50 µm. (I, J, K, L, M, N, O, P, Q, R, S, T, and U) Comparison of gene expression levels of *Pparg2* (I), *Adipoq* (J), *Lpl* (K), *Gabrg2* (L), *Sncg* (M), *Pdgfra* (N), *Tbx1* (O), *Cd137* (P), *Plf* (Q), *Pdgfrb* (R), *Ly6c*1 (S), *Cd9* (T), and *Wt1* (U) mRNA between epididymal SPA and inguinal SPA. All values represent mean ± s.e. (*n* = 4) of relative expression levels of mRNA (Epididymal as 1).

**Figure 4:**
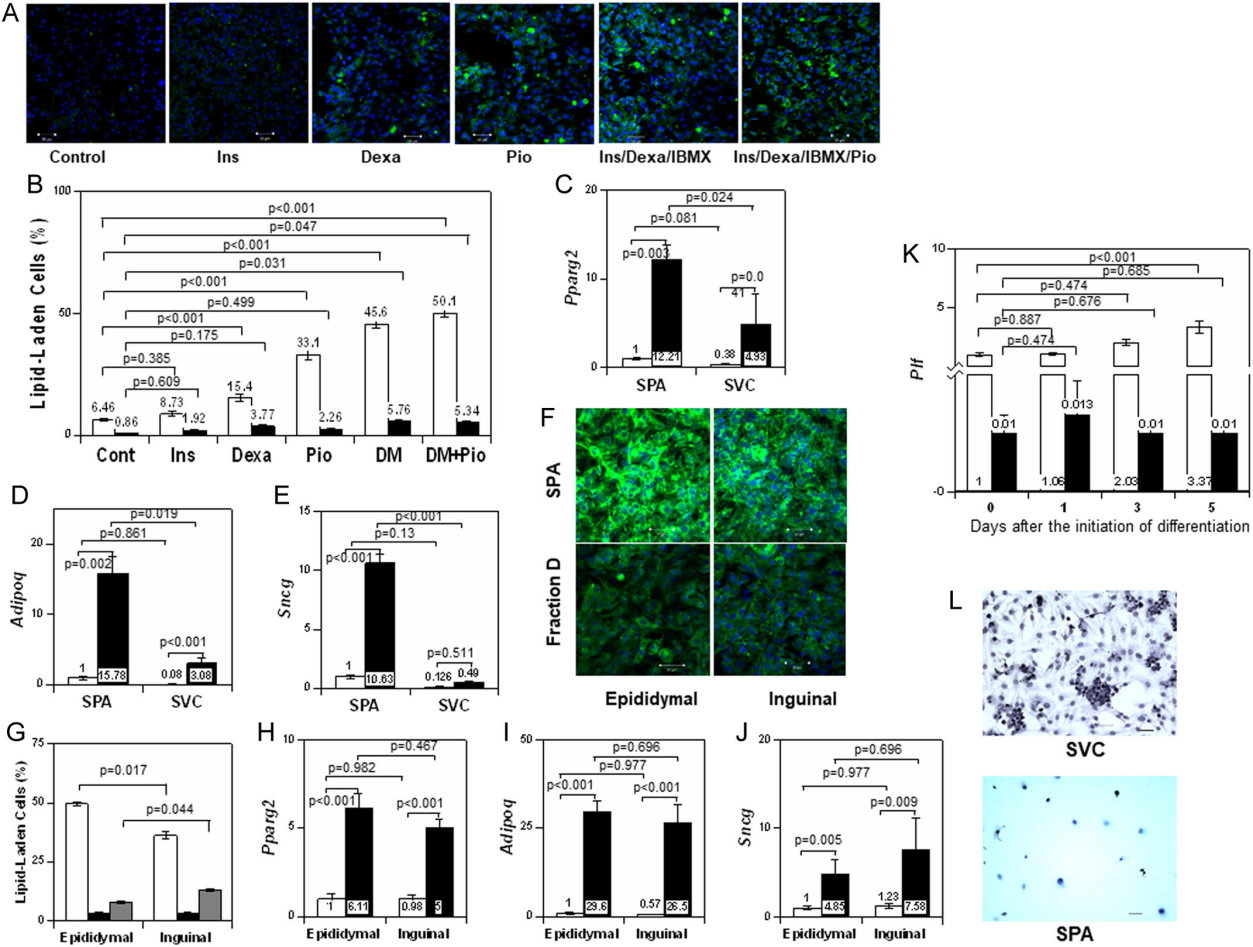
Differentiation of SPA into mature adipocytes. (A) SPA were treated without (control) or with 100 nM insulin (Ins), 1 µM dexamethasone (Dexa), 10 µM pioglitazone (Pio), differentiation medium (Ins/Dexa/IBMX) containing 200 nM insulin/1 µM dexamethasone/0.5 mM IBMX or 10 µM pioglitazone and differentiation medium (Ins/Dexa/IBMX/Pio) for 5 days. Differentiation was evaluated by accumulation of lipid stained with LipiDye (green). (B) Quantification of the results shown in (A). Percentages of lipid-laden cells in SPA (open column) and SVC (closed column). The *P* values are for each control value (*n* = 4). (C, D, and E) Effects of treatment with differentiation medium for 5 days in SVC and SPA on the expression of *Pparg2* (C), *Adipoq* (D), and *Sncg* (E). Open column: control cells, Solid column: treated cells, (*n* = 4). (F and G) Comparison of ability to differentiate into lipid-laden cells between epididymal and inguinal SPA/fraction D. Epididymal and inguinal SPA/fraction D were incubated with the differentiation medium for 5 days. Image of typical lipid-laden cells in differentiated SPA (F) and quantified results (G) were shown. Open column: SPA, Solid column: SVC, Gray column: fraction D (*n* = 3). (H, I, and J) Differentiation was evaluated with expression levels of *Pparg2* (H), *Adipoq* (I), and *Sncg* (J). Open column: control cells, Solid column: differentiated cells, (*n* = 3). (K) Time course of *Plf* expression levels in SPA (open column) and SVC (solid column) during adipogenic differentiation (*n* = 3). (L) Ability of SPA and SVC to differentiate into osteoblasts. SPA and SVC were induced to differentiate into osteoblasts. Differentiation was evaluated by alkaline phosphatase staining (black).

**Figure 5:**
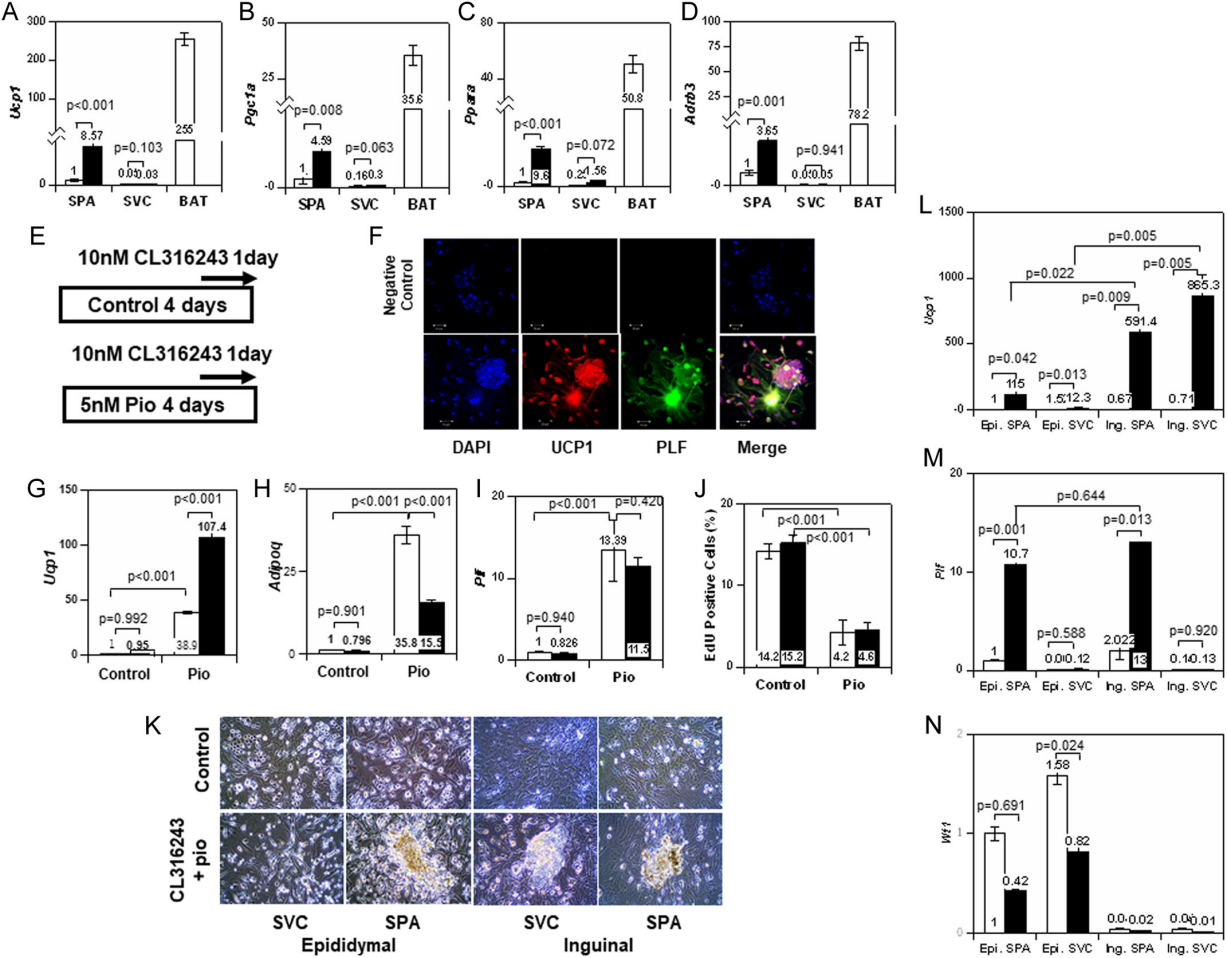
Expression of PLF, UCP1, and mitochondria-related genes in differentiated SPA and differentiated SVC. (A, B, C, and D) Results of real-time PCR were demonstrated. (A)* Ucp1*, (B) *Pgc1a*, (C) *Ppara*, and (D) *Adrb3*. All values represent the mean ± s.e. (*n* = 8) of relative expression levels of mRNA (SPA, control as 1). To compare the expression levels of these genes, the relative expression levels in BAT were added. Open column represents control cells and solid column represents differentiated cells with the differentiation medium and 10 µM pioglitazone for 5 days (*n* = 4). (E) Protocol for treatment with pioglitazone (Pio) and CL316243. (F) Expression of PLF and UCP1 in SPA treated with Pio and CL316243. Scale: 50 µm. (G-I) Relative expression levels of *Ucp1* (G), *Adipoq* (H), and* Plf* (I) in SPA treated with or without Pio and CL316243 are shown. Open column represents results in the absence of CL316243 and solid column represents those in the presence of CL316243. All values represent the mean ± s.e. (*n* = 4) of relative expression levels of mRNA (control without CL316423 as 1). (J) Effect of treatment with CL316243 and Pio on cell growth in SPA. Percent of EdU-positive cells was shown. Open column: Control, Solid column: CL316243 (*n* = 3). (K) Images of cultured epididymal SPA, epididymal SVC, inguinal SPA, and inguinal SVC treated with or without Pio and CL316243. (L–M) Expression levels of *Ucp1* (L), *Plf* (M), and *Wt1* (N) mRNA in with (treated: solid) or without (control: open) CL316243 and Pio in epididymal and inguinal SPA and SVC (*n* = 3).

**Figure 6:**
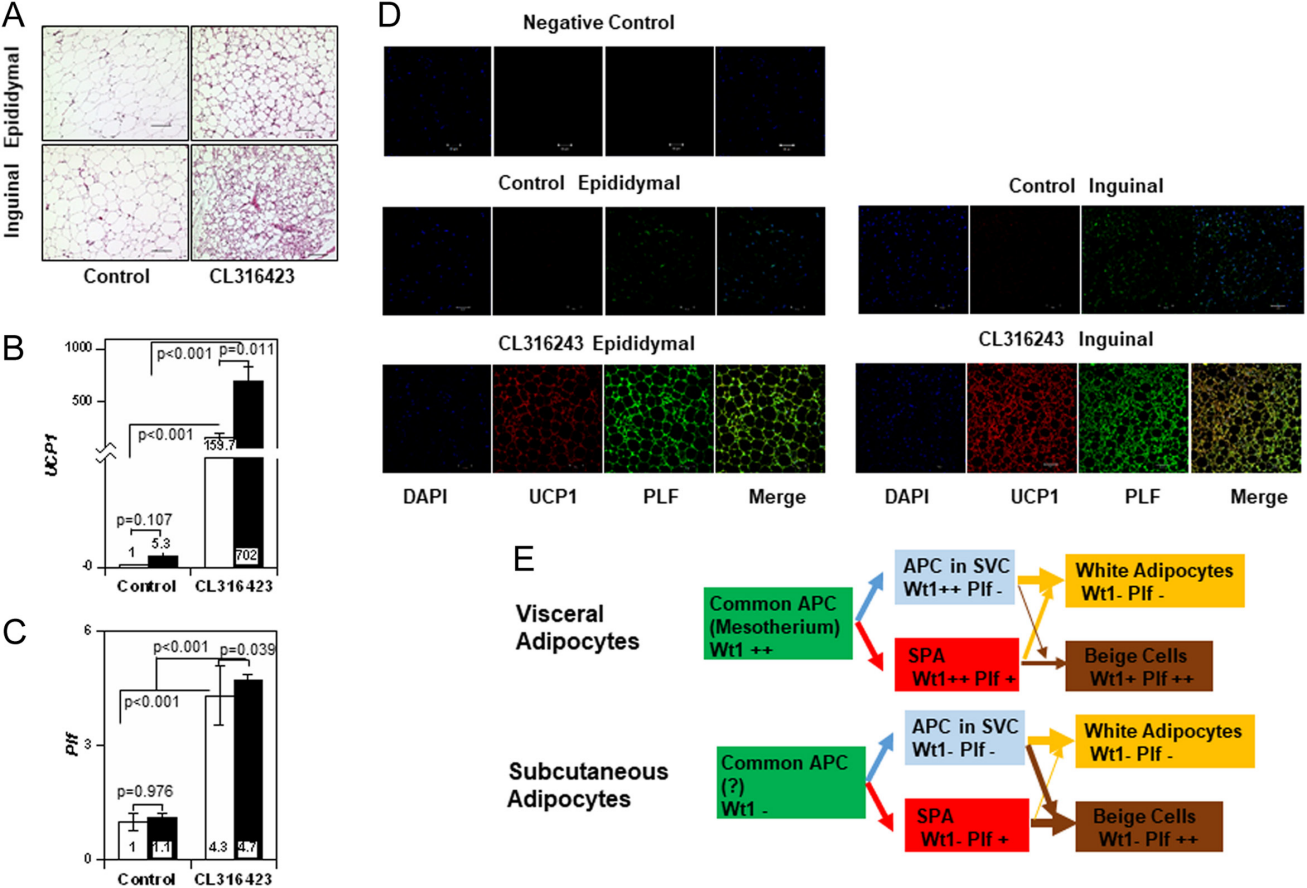
Expression of UCP1 and PLF in beige cells. (A) Beige cells were generated by the daily peritoneal injection of CL316243 to C57/BL mice for 7 days of a total 21 days. HE staining of specimens obtained from epididymal and inguinal fat isolated from control or treated mice (*n* = 4) is shown. (B and C) Expression of* Ucp1*(B) and *Plf* (C) mRNA in epididymal (open) and inguinal (solid) fat is shown (control in epididymal adipose tissue as 1, *n* = 4). (D) Expression of UCP1 and PLF in epididymal and inguinal fat isolated from control or treated mice with CL316243. Scale: 50 µm. (E) A model for differentiation of SPA and adipose progenitor cells (APC) in visceral and subcutaneous adipose tissues.

All experimental data were shown as means ± s.e.m. (*n*, sample numbers). Statistical comparisons were performed using Excel Statistics with an unpaired two-tailed Student’s *t*-test (Figs 1, 2 and 3) and Kruskal-Wallis one way ANOVA (Figs 4, 5 and 6). Significance was defined as *P* < 0.05.

## Results

### Isolation of SPA

Sugihara *et al*. found that ceiling culture, the method to adhere floating mature adipocytes on the bottom upside down, provides a clear image of mature white adipocytes (Sugihara *et al.* 1987) (Supplementary Fig. 1A). We noted that round or spindle shaped cells sank to the bottom of the flask when ceiling culture was performed at the first step (Supplementary Fig. 1B). Furthermore, these cells were able to incorporate BrdU, indicating that many non-unilocular cells are present in the floating cell fraction of adipose tissue. Based on these observations, we aimed to obtain an SPA-rich fraction with centrifugation. As shown in Fig. 1A, dispersed cells obtained by collagenase digestion of the adipose tissue were separated into fraction A, fraction B, and fraction C by centrifugation. Obviously, fraction A and C correspond to mature white adipocytes and SVC, respectively. In addition, fraction D was obtained by centrifugation of dispersed cells at 226 ***g*** for 3 min, which was regarded as a total non-adipocyte cell fraction for flow cytometry.

The shape of cultured cells in fraction B was small, round, spindle-like or linear, while cells in SVC had a broad, spindle or polygonal shape (Fig. 1B). As the cell numbers increased, linear cells in fraction B become wider. When cultured for about 10 days, the morphological difference between fraction B and SVC decreased; however, small round cells remained in fraction B. As shown in Supplementary Fig. 1C, often small round cells were observed to form clusters around the linear cells. Of the cultured cells, 85 to 95% in fraction B were positive for adiponectin and leptin, in contrast to fewer than 10% of cells in SVC (Fig. 1C). These cells expressed adiponectin and leptin regardless of their morphology (Supplementary Fig. D). Time-lapse image of cells in fraction B displayed cell division with the form of some cells changing (Supplementary Fig. 1E). Cell numbers of fraction B and SVC increased similarly (Supplementary Fig. 1F). Calculated doubling time of cells in fraction B and SVC was 38.5 ± 7.7 h and 29.8 ± 2.3 h (*P* = 0.479), respectively. Immunoblot analysis detected adiponectin, leptin, and PPARγ cells in fraction B (Fig. 1D and E). Incorporation of EdU, an analog of thymidine, was detected in adiponectin-labeled cells (Fig. 1F). The percentage of EdU-positive cells was not different between SVC and cells in fraction B (Fig. 1G). These results indicate that many cells in fraction B exhibited adipocyte-specific proteins and the ability to proliferate, which are hallmarks of SPA. Therefore, fraction B was regarded as an SPA-rich fraction.

### Characterization of SPA

Next, we explored the characteristics of SPA. Gene expression of SPA and SVC was examined comprehensively by microarray (GEO accession number: GSE110886). In the comparison between SVC and SPA and between SPA and MWA, we studied genes with more than five-fold differences in expression levels. As shown in Table 1, several genes specific for adipocytes, including adiponectin (*Adipoq*), leptin (*Lep*), and resistin (*Retn*), and the expression levels of the genes abundantly expressed in adipocytes, including lipoprotein lipase (*Lpl*), and fatty acid binding protein 4 (*Fabp4*) were higher in SPA than in SVC. Interestingly, neuronal genes, such as synuclein γ (*Sncg*), 5-hydroxytryptamine receptor 1F (*Htr1f*), gamma-aminobutyric acid type A receptor gamma 2 (*Gabrg2*), were abundantly expressed in SPA. In addition, expression levels of members of the prolactin family, prolactin family 2, subfamily c, member 3 (*Prl2c3: Plf*) and prolactin family 2, subfamily c, member 5 (*Prl2c5*) were elevated only in SPA. On the other hand, microarray revealed that numerous adipocyte-specific genes, such as *Reten*, *Adipoq*, *Plin1*, and *Plin4*, and genes abundantly expressed in white adipocyte, including *Klb*, *Lpl*, *Ppara*, *Fabp4*, and *Ppargc1a*, were more expressed in MWA than in SPA (Table 2). Several neuronal genes, such as *Adora1*, *Sncg*, and* Syn2*, were more abundantly expressed in MWA than in SPA. Many of the genes listed in Table 1 were also listed in Table 2 (shown in red in Table 2).

**Table 1:** Genes five times more up-regulated in SPA compared with SVC determined by microarray.

Adipocyte-specific genes	*Retn*, *Adipoq*, *Adig*, *Cfd*, *Adrb3*, *Aqp7*, *Plin1*, *Plin4*, *Lep*, and *Rbp7*
Genes expressed in WAT abundantly	*Cyp2el*, *Car3*, *Slc7a10*, *Thrsp*, *Serpina3c*, *Bche*, *Igfbp5*, *Pck1*, *Rasd1*, *Fabp4*, *Acvr1c*, *Lpl*, *Lipe*, *Aox1*, and *Angptl4*
Neuronal genes	*Ecel1*, *Prlr*, *Olfr558*, *Ednrb*, *Olfr986*, *Olfr630*, *Mroh8*, *Htr1f*, *Cbln3*, *Rasd1*, *Sncg*, *Syn3*, *Snca*, *Robo2*, *Tas2r118*, *Olfr670*, *Bche*, *Gabrg2*, *Slc6a15*, *Pcdh17*, *Hpca*, and *Nrcam*
Prolactin family-genes	*Prl2c3 (Plf)* and *Prl2c5*

**Table 2:** Genes five times more up-regulated in MWA compared with SPA determined by microarray.

Adipocyte-specific genes	*Retn*, *Adipoq*, *Adig*, *Plin4*, *Plin1*, *Adrb3*, *Aqp7*, and *Pparg*
Genes expressed in WAT abundantly	*Pck1*, *Ces1d*, *B3galt2*, *Pp1r1a*, *Gpihbp1*, *C1dec*, *Abcd2*, *Rbp7*, *Steap4*, *Chp2*, *Klb*, *Ffar4*, *Il6*, *Fabp9*, *Ffar2*, *Ucp1*, *Agt*, *Fabp4*, *Ptger3*, *Ppara*, *Ppargc1a*, *Irs2*, *Adipor2*, *Inhbb*, *Ebf3*, and *Lpl*
Neuronal genes	*Atp1a2*, *Nnat*, *Adora1*, *Ntsr2*, *Sncg*, *Hpca*, *Oxtr*, *Ncan*, *Negr1*, *Syn2*, *Ntm*, and *Sytl3*

We confirmed the expression levels of adipocyte genes and neuronal genes listed in both Tables 1 and 2 in SVC, SPA, and MWA with real-time PCR. As expected from the results of microarray comparing among SVC, SPA, and MWA, mRNA levels of adipocyte-specific or related genes, *Pparg2*, *Adipoq*, *Lep*, *Retn*, *Plin1*, and* Plin4*, were increased in the order of SVC < SPA << MWA (Fig. 2A, B, C, D, E and F). Other important adipocyte genes, such as *Fabp4*, *Adrb3*, *Klb* and* Aqp7*, showed the same expression pattern as these genes (Fig. 2G, H, I and J). The expression levels of genes involved in triglyceride synthesis, *Srebp1* and *Fas*, were SVC = SPA < MWA (Fig. 2K and L). Neuronal gene, *Sncg* and *Gabrg2* were increased in the order of SVC < SPA < MWA as shown by microarray (Fig. 2M and N). In contrast, *Plf* was predominantly expressed in SPA (Fig. 2O). Next, we evaluated the expression levels of progenitor cell markers. Markers of adipocyte progenitor, *Dlk1*, *Pdgfra* and *Pdgfrb*, were expressed in SPA, as well as in SVC (Fig. 2P, Q and R).* Ly6c1* and *CD9*, regarded as markers of fibro-inflammatory phenotype in the stromal vascular fraction (Marcelin *et al*. 2017, Hepler *et al*. 2018), were less abundantly expressed in SPA than in SVC (Fig. 2S and T). No significant differences in the expression levels of markers of brown adipocyte progenitors, *Zic1* and *Myf5*, were detected between SVC, SPA, and MWA (Fig. 2U and V). Since the expression levels of these genes in SVC, SPA, and MWA were very low compared with those in BAT, they were not considered meaningful. However, markers of beige cell progenitors, *Cd137* and* Tbx1*, were expressed predominantly in SPA (Fig. 2W and X). These results suggested that SPA may have properties of beige cell progenitors.

Immuno-cytochemical studies showed that cultured SPA express SNCG (Fig. 3A) and PLF (Fig. 3B). Since the obtained images showed morphological diversity of SPA, we further analyzed this with FCM using antibodies against cell surface antigens. As shown in Fig. 3D, AQP7^+^, ADRB3^+^ clusters were detected (red spots) as well as AQP7^-^, ADRB3^+^ (green spots) clusters in fraction D cells (SVC + SPA), compared with the negative control (Fig. 3C). AQP7^+^, ADRB3^+^ cells were widely distributed throughout the examined cells (Fig. 3E). Moreover, with respect to cell diameter, these cells did not show any particular distribution among the examined cells (Fig. 3F). Very few AQP7^+^, ADRB3^+^ cells or AQP7^-^, ADRB3^+^ cells were detected in either the epididymal or inguinal fraction C (SVC) (Supplementary Fig. 2B and D), whereas similar percentages of AQP7^+^, ADRB3^+^ cells and AQP7^-^, ADRB3^+^ cells were detected in epididymal and inguinal fraction B (SPA) (Supplementary Fig. 2C and E) compared with negative control (Supplementary Fig. 2A and F). These results supported the morphological diversity of SPA. The diameter of SPA determined by FCM was 3–18 µm, which coincided with the value measured under the microscope (Supplementary Fig. 2G).

Immuno-histochemical studies in adipose tissues supported the results that SNCG were expressed in mature white adipocytes and non-mature white adipocytes expressing adiponectin (Fig. 3G). In contrast, PLF was expressed only in adiponectin-labeled non-mature white adipocytes, that is, SPA in adipose tissue (Fig. 3H).

Next, we compared the characteristics of epididymal SPA with inguinal SPA. Statistically significant differences in gene expression levels were not detected in adipocyte or neuronal genes (Fig. 3I, J, K, L and M). Expression levels of *Pdgfra*, *Tbx*1, and *Plf* , but not *Cd137*, were greater in inguinal SPA than epididymal SPA (Fig. 3N, O, P and Q). In addition, *Ly61c* and *Cd9*, but not *Pdgfrb*, were more abundantly expressed in inguinal SPA than epididymal SPA (Fig. 3R, S and T). The expression level of *Wt1* was markedly suppressed in inguinal SPA compared with epididymal SPA (Fig. 3U). Immuno-histological examination showed the number of PLF-positive cells to be higher in inguinal adipose tissue than in epididymal tissue, with both decreasing with weeks of age (Supplementary Fig. 2H and I). These results suggested that the approximate number of SPA in epididymal fat did not differ from that in inguinal fat.

### Ability of SPA to differentiate into adipocytes

To determine whether SPA are adipose progenitor cells, SPA were cultured in DMEM containing 10 µM pioglitazone, 100 nM insulin, 1 mM dexamethasone or the adipogenic differentiation medium containing insulin (200 nM)/dexamethasone (1 µM)/IBMX (0.5 mM) for 5 days. A few lipid droplets were seen in SPA cultured in DMEM as a control. Indeed, the differentiation medium induced conversion of SPA to lipid-laden cells (Fig. 4A). Treatment with dexamethasone and pioglitazone alone increased the number of lipid-laden cells in SPA, but not in SVC. Stimulation with differentiation medium plus pioglitazone resulted in maximal differentiation in both SPA and SVC (Fig. 4A and B). Lipid accumulation was particularly observed in clusters of small cells (Supplementary Fig. 3A). This finding suggested that the cell population shown in Supplementary Fig. 1C differentiates more easily into lipid-laden cells. Expression levels of* Pparg2* and *Adipoq* were increased by the induction of differentiation with the differentiation medium in both SPA and SVC (Fig. 4C and D). A significant increase in *Sncg* was detected only in SPA (Fig. 4E).

Incubation with differentiation medium resulted in fewer lipid-laden cells in inguinal SPA than epididymal SPA (Fig. 4F and G), whereas the percentage of lipid-laden cells was lower in differentiated epididymal fraction D than differentiated inguinal fraction D. This reversal may have been observed because the number of SPA was larger in inguinal adipose tissue than in epididymal adipose tissue as shown in Supplementary Fig. 2I. However, no differences were detected in the expression levels of* Pparg2*, *Adipoq*, or *Sncg* between the differentiated inguinal SPA and differentiated epididymal SPA (Fig. 4H, I and J). These results indicated that, although epididymal SPA might differentiate more easily into adipocytes than inguinal SPA, the difference is slight.

Time-course study revealed that the expression of *Plf* in SPA was significantly increased on day 3 after the induction of differentiation, whereas it was never increased in the differentiation process in SVC (Fig. 4K). As a result, both SPA and SVC could differentiate into adipocytes; however, SVC differentiated without ever becoming SPA. Immuno-cytochemical study confirmed expression of PLF in both SPA and differentiated SPA (D-SPA), but not in SVC or differentiated SVC (D-SVC) (Supplementary Fig. 3B). Moreover, PLF was more abundantly detected in D-SPA than in SPA when observed under the same conditions. Therefore, the increased expression levels of *Plf* mRNA in D-SPA shown in Fig. 4K were considered to be due to elevated PLF expression in D-SPA.

In addition, we evaluated the ability of SVC and SPA to differentiate into osteoblasts. SVC and SPA were incubated with RPMI/10% FBS containing an osteogenic cocktail of L-ascorbic acid, hydrocortisone acid, and β-glycerophosphoric acid (Takara Bio). The medium was changed every 4 days. After the treatment for osteogenic induction for 15 days, cells were stained with alkaline phosphatase using the TRACP & ALP double-stain kit (Takara Bio). SVC, but not SPA, differentiated into alkaline phosphatase-positive osteoblast like cells (Fig. 4L). These results indicated that SPA lose multipotency compared with SVC, suggesting that SPA may be cells in the stage of intermediate differentiation.

Mouse age did not affect the ability of SPA to differentiate into adipocytes as assessed by both lipid-laden cells (Supplementary Fig. 3C) and expression levels of adipocyte-specific genes, *Pparg2* and *Adipoq* (Supplementary Fig. 3D and E).

### Characterization of differentiated SPA

Next, we studied the ability of SPA and SVC to differentiate into beige cells. Real-time PCR showed that the mRNA level of *Ucp1* was increased after the adipogenic differentiation in SPA, but not in SVC (Fig. 5A). Genes involved in heat production, *Pgc1a*, *Ppara*, and* Adrb3*, were up-regulated in SPA during differentiation (Fig. 5B, C and D), although the levels were low compared with those in BAT. Immuno-cytochemical study revealed that UCP1 was expressed in D-SPA (Supplementary Fig. 4A). The expression of UCP1 protein in D-SPA, but not in SPA, was confirmed by immunoblot analysis (Supplementary Fig. 4B and C). The results showed that PLF was expressed in D-SPA, but not in mature white adipocytes as shown in Fig. 3B, clearly indicating that D-SPA differ from mature white adipocytes.

Since beige cells were induced by β3-adrenergic receptor agonist (CL316243), we studied whether stimulation with CL316243 would lead to differentiation of cultured SPA into beige cells. Although treatment with CL316243 alone did not cause morphological change or gene expression, pretreatment with pioglitazone and additional treatment with CL316243 (Fig. 5E) generated cell clusters in SPA (Supplementary Fig. 4D). PLF was detected in the UCP1-positive cells (Fig. 5F). The expression level of *Ucp1* mRNA was increased by the treatment with pioglitazone and further enhanced by the addition of CL316243 to SPA (Fig. 5G). Treatment of SPA with pioglitazone and CL316423 up-regulated *Ucp1* expression level more markedly than treatment with insulin/dexamethasone/IBMX. These results suggested that additional treatment with CL316243 promoted differentiation into beige-like cells in SPA. In contrast, the increased expression level of *Adipoq* by pioglitazone was suppressed by CL316243 in SPA (Fig. 5H), while that of *Plf* was not affected (Fig. 5I). Although incubation with pioglitazone reduced incorporation of EdU, CL316243 did not affect the incorporation of EdU (Fig. 5J). The potency of SPA to differentiate into beige cells was decreased at the age of 20 weeks (Supplementary Fig. 4E).

Next, we assessed the ability of SVC and SPA derived from inguinal and epididymal fat to differentiate into beige cells. Treatment with pioglitazone and CL316243 resulted in the formation of packed cell clusters in inguinal SVC and SPA as well as epididymal SPA, but not epididymal SVC (Fig. 5K). Expression levels of *Ucp1* mRNA in differentiated inguinal SVC and SPA markedly exceeded that of differentiated epididymal SPA (Fig. 5L). These results indicated that SVC and SPA derived from inguinal fat were more capable of differentiating into beige-like cells than SPA from epididymal fat. Increased expression of *Plf* with differentiation was comparable between epididymal and inguinal SPA (Fig. 5M). Expression of *Wt1* decreased to about half in both epididymal SPA and SVC by differentiation (Fig. 5N).

### Expression of PLF in beige cells

To confirm these results, beige-like cells were generated by pharmacological stimulation of β3-adrenergic receptor *in vivo*. Cells with apparent characteristics of beige cells were detected in inguinal and epididymal fat after the daily intraperitoneal injection of CL316243 for 7 days. Clusters of multilocular cells, characteristic of beige cells, were frequently observed in the inguinal fat, while small clusters were also observed in the epididymal fat isolated from CL316243-treated mice (Fig. 6A). Expression of *Ucp1* mRNA increased in both inguinal and epididymal fat immediately after the CL316243 injection, but the increase in the former was greater. The increased *Ucp1* mRNA level was still present 1 week after the injection in inguinal, but not epididymal, fat (Fig. 6B). Conversely, *Plf* mRNA levels were increased similarly after the injection of CL316243 in epididymal and inguinal fat (Fig. 6C). These results were supported by the measurement of protein levels of UCP1 and PLF (Supplementary Fig. 4F, G and H). Immuno-histochemical study demonstrated that UCP1 was expressed in both epididymal and inguinal fat immediately after the CL316243 injection, although the expression pattern was different (Fig. 6D). In inguinal fat, the expression of PLF and UCP1 did not always coincide, whereas PLF was detected consistently with UCP1 in epididymal fat. These results suggested that epididymal beige cells might be derived from PLF-positive SPA, with inguinal beige cells derived from PLF-positive SPA and PLF-negative SVC.

## Discussion

We previously identified SPA as a novel population of cells expressing both adipocyte-specific genes and proliferative activity in adipose tissue (Kajita *et al.* 2013). In this study, we focused on the characteristics of SPA. First, we attempted to isolate SPA from other non-adipocyte cells (SVC). We found that inverted ceiling culture, as shown in Supplementary Fig. 1B, yielded SPA-like cells. Then, we tried to collect these sedimentary cells by centrifugation. As expected, almost all of the cells cultured in fraction B were positive for adiponectin. A time-lapse study showed cell division in these cells. Moreover, incorporation of EdU was detected in the adiponectin-positive cells, allowing us to conclude that the cells in fraction B consist of SPA.

Next, we investigated the gene expression of SPA as compared with that of SVC and MWA. A microarray was performed between SVC and SPA and between SPA and MWA to determine the general trend, and then quantitative analysis was performed by real-time PCR. A microarray analysis revealed that the expression levels of adipocyte-specific genes, such as *Adipoq*, *Lep*, *Retn*, and those expressed abundantly in adipocytes, including *Lpl* and *Fabp4*, were increased in SPA as compared with in SVC. Several of these genes were also more abundantly expressed in MWA than in SPA. Data obtained by real-time PCR confirmed that levels of genes characteristically expressed in adipocytes were increased in SPA and even more so in MWA. These results suggest that SPA may be intermediate cells in the course of adipogenesis.

We measured the expression of genes considered to be markers of APC. In this way we were able to document that the APC markers, *Dlk1*, *Pdgfra* and *Pdgfrb*, were expressed in SVC and SPA as well. These results suggested that SPA may make up one type of APC. Expression levels of *Ly6c1* and *Cd9*, markers of fibro-inflammatory progenitors (Marcelin *et al*. 2017, Hepler *et al*. 2018), in SPA were lower than in SVC. As a result, SPA was expected to be differentiated into white adipocytes. In addition, markers of beige cell progenitors, *Cd137* and *Tbx1* (Seal *et al.* 2008, de Jong *et al.* 2015), were more markedly expressed in SPA than in SVC. These results suggested that SPA may also be a progenitor of beige cells.

The existence of SPA was also proved by FCM. SPA, which were detected as AQP7^+^, ADRB3^+^ cells, displayed a morphologically wide distribution. Very few AQP7^+^, ADRB3^+^ and AQP7^-^, ADRB3^+^ cells were detected in both epididymal and inguinal fraction C (SVC), whereas they were observed to the same extent in epididymal and inguinal fraction B (SPA). Since the shape of SPA varied greatly under microscopic observation, the results of FCM shown in Fig. 3E and F confirmed the morphological heterogeneity of SPA. Our data showed that expression of adipocyte-specific genes and proliferative activity were independent of their morphology. However, the presence of AQP7^+^, ADRB3^-^ cells revealed that SPA are not homogenous in terms of gene expression. Moreover, as shown in Supplementary Fig. 3A, cell clusters formed by small round cells more easily differentiate into lipid-laden cells, suggesting the functional heterogeneity of the corresponding cell morphology.

The percentages of AQP7^+^, ADRB3^+^ and AQP7^-^, ADRB3^+^ cells in fraction B shown in Supplementary Fig. 2F did not agree with the results from microscopic observation that 85–95% cells in fraction B were positive for adiponectin and leptin. This may be due to a problem with FCM sensitivity due to weak SPA antigen signals. Furthermore, as shown in Supplementary Fig. 1C, small cells forming clusters, which were equipped with the characteristics of SPA, were often detected in SPA. However, in FCM, these cells are excluded before measurement or not correctly evaluated as individual cells. The discrepancy between FCM and microscopic observation might be explained by these causes.

In this study, treatment with the differentiation medium promoted adipogenic differentiation in both SVC and SPA. Furthermore, SPA, but not SVC, were shown to differentiate into lipid-laden cells expressing adipocyte-specific genes when treated with pioglitazone. SPA were thus considered to be more sensitive to these agents than APC in SVC. These results might be attributable to the lower amounts of the fibro-inflammatory progenitor markers *Ly6cl* and *Cd9* expressed in SPA compared with SVC (Marcelin *et al*. 2017, Hepler *et al*. 2018). The fact that D-SPA possessed lipid droplets and expressed increased levels of adipocyte-specific genes indicated that they were closer to white adipocytes in the differentiation stage than SPA. However, expression of PLF indicates that D-SPA are different from mature white adipocytes. These findings together indicate that D-SPA are still in the stage of mature white adipocyte progenitors. In addition, the absence of increased mRNA of *Plf* expression during SVC differentiation implied that SPA and SVC follow different pathways to differentiation.

Since* Cd137* and *Tbx1*, markers of beige cells, were abundantly expressed in SPA, we examined the possibility that SPA might be a progenitor of beige cells. We found that *Ucp1* and other genes related to heat production, *Pgc1a*, *Ppara* and *Adrb3*, were up-regulated during adipogenic differentiation. Then, we determined whether or not treatment with a β3-adrenergic receptor agonist promoted browning of SPA and demonstrated that pioglitazone and CL316243, in tandem, converted SPA into cell clusters resembling beige cells.

Next, we studied the gene expression and ability of epididymal and inguinal SPA to differentiate into lipid-laden cells and beige cells. There were no differences between epididymal and inguinal SPA in the expression levels of adipocyte genes and neuronal genes. *Plf* was greatly expressed in inguinal SPA compared with epididymal ones. Several progenitor markers, *Pdgfra*, *Tbx1*, *Ly6c1* and *Cd9*, were more abundantly expressed in inguinal SPA than in epididymal ones, whereas *Wt1*, a marker of visceral adipocytes (Chau *et al*. 2014), was markedly expressed in epididymal SPA. In comparing the differentiation potential of epididymal and inguinal SPA into lipid-laden cells, the former was better when evaluated by fat accumulation, while there was no difference when evaluated by expression of adipocyte genes. Therefore, we concluded that epididymal SPA are slightly more capable of differentiation into white adipocyte than inguinal SPA. This may be related to the fact that *Ly6c1* and *Cd9* were less expressed in epididymal SPA. On the other hand, differentiation capacity into beige-like cells was greater in inguinal SPA than in epididymal ones. The fact that *Tbx1*, a marker of beige cell progenitor, was greatly expressed in inguinal SPA may explain this difference.

We confirmed that PLF was expressed abundantly in CL316243-induced beige cells in epididymal fat. However, a quantitative study by real-time PCR and immunoblot showed that the relation between UCP1 and PLF differed between epididymal and inguinal fat. The expression level of UCP1 was higher in inguinal than in epididymal fat, whereas PLF was expressed equally in both tissues immediately after the injection of CL316243. An immuno-histochemical study showed that the expression of UCP1 and PLF was consistent in epididymal, but not inguinal, fat. In inguinal fat, cells expressing UCP1, but not PLF, were observed. These results suggest that both SPA and SVC might be precursors of beige cells in inguinal fat, while only SPA might be the source of beige cells in epididymal fat. This contention is supported by the results shown in Fig. 5L and M.

Our results indicated that epididymal and inguinal SPA expressed both *Pdgfrq* and *Pdgfrb*. A recent study indicated that PDGFRα signaling generates beige cells, while PDGFRβ generates white adipocytes (Gao *et al.* 2018). Interestingly, Lee *et al*. reported that PDGFRα^+^ cells exhibited the ability to differentiate into both white adipocytes and beige cells (Lee *et al*. 2012). They also demonstrated that PDGFRα^+^ cells showed proliferating activity and expression of the adipocyte-specific gene, *Plin1*. On the other hand, Hepler *et al.* demonstrated that cell cluster characterized by LY6C^-^ CD9^-^ PDGFRβ^+^ in stromal vascular cells expressed genes typically characteristic of mature adipocytes, such as *Adipoq*, *Retn*, and *Adrb3*. We are not yet able to speculate about the role of PDGFR signals in SPA, and further research will be required to define it.

Most of the experiments in this study were performed using mice at 10 weeks of age. The percentage of PLF-positive cells, which were regarded as SPA, decreased at 20 weeks of age. Age did not affect the ability of SPA to differentiate into white adipocytes, although the potency to differentiate into beige cells decreased at 20 weeks of age. These results suggested that the ability to produce new white adipocytes and beige cells declines with age.

Taken together, the role of SPA in adipogenesis is shown in Fig. 6E. Since Wt1 is regarded as a marker of visceral adipocyte, progenitors derived from mesothelium, epididymal (visceral) SPA are considered to have a different origin from that of inguinal (s.c.) SPA. A recent study identified PRX1 as a marker of s.c. adipose progenitor (Krueger *et al*. 2014, Sanchez-Gurmaches *et al*. 2015). On the other hand, PDGFRα, PDGFRβ, and DLK1 are expressed in both adipose progenitors (Hudak *et al*. 2014, Lee *et al*. 2015, Vishvanath *et al.* 2015, Gulyaeva *et al*. 2018). In the same way, PLF is considered as a common marker of SPA in s.c. and visceral fat.

The result shown in Fig. 4K indicated that SPA and APC in SVC differentiate into white adipocytes and beige cells in parallel. The significance of the differentiation of the two pathways into visceral and s.c. adipocytes, respectively, needs to be elucidated. Moreover, it was documented that SPA are heterogeneous and that inguinal SPA abundantly expressed beige cell marker (Tbx1) compared with epididymal SPA. Whether the SPA in s.c. adipose tissue and visceral adipose tissue are uniform as a progenitor for white adipocytes and beige cells, or they contain different progenitors for each cell types, is an issue for future consideration.

## Conclusion

Our research demonstrated that SPA express several adipocyte-specific genes, neuronal genes, and genes for the prolactin family including PLF. SPA differentiate into lipid-laden cells when stimulated with the adipogenic differentiation medium containing insulin/dexamethasone/IBMX. Since D-SPA express UCP1 and PLF, which are also detected in beige cells, but not in mature white adipocytes, SPA, especially s.c. SPA, are considered to be progenitors of beige cells.

## Supplementary Material

Supplementary Methods

Suppl. Table 1 Utilized antibody list.

Suppl. Table 2 Oligonucleotide primers designed based on sequences from the GenBank database.

Suppl. Fig. 1 (A) Ceiling culture (upper panel) and cultured cells (lower panel). Floating cells isolated from five C57/BL mice were applied. Cultured cells were treated with BrdU (10 μM) for 12 h. Incorporated BrdU was visualized using anti-BrdU antibody. (B) Reverse ceiling culture (upper panel) and cultured cells (lower panel). (C) Image of small round cells forming cell clusters around linear cells observed in fraction B. (D) Immuno-cytochemistry of SPA by enzymatic method. (E) Time-lapse image of cultured cells obtained in Fraction B (SPA). Interval: 1 hr. Arrows represent proliferating cells. (F) Growth analysis of cells in fraction B and SVC: Cells in fraction B (red) and SVC (black) were seeded on 96 well plate. Numbers of nuclei stained by DAPI were counted

Suppl. Fig. 2 (A-E) Flow cytogram of AQP7+; ADRB3+ cells in negative control (A), epididymal SVC (B), epididymal SPA (C), inguinal SVC (D) and inguinal SPA (E). (F) Percentage of AQP7-; ADRB3- cells (white), AQP7+; ADRB3- cells (blue), AQP7+; ADRB3+ cells (black) and AQP7-; ADRB3+ cells (red) were shown (n=4) (G) Measurement of SPA and MWA cell diameters under a microscope. Red: PLF, Blue: DAPI, White letters and green letters represent SPA and MWA cell diameter, respectively. (E) Expression of PLF in epididymal and inguinal adipose tissue. (I) Percentage of PLF-positive cells/total cells in epididymal adipose tissue (open) and inguinal (solid) isolated from mice of 5 weeks, 10 weeks and 20 weeks of age. PLF-positive cells were determined as number of nuclei (blue) surrounded by PLF (red) and total cells as number of nuclei (n=3).

Suppl. Fig. 3 (A) Typical image displaying clustered round cells that were easily differentiated into lipid-laden cells. (B) Expression of PLF in SPA, SVC, differentiated SPA (D-SPA) and differentiated SVC (D-SVC) (C) Lipid-laden cells in adipogenic differentiated epididymal SPA isolated from mice of 5 weeks, 10 weeks and 20 weeks of age. (D, E) Expression of Pparg2 (D) and Adipoq (E) mRNA in adipogenic differentiated epididymal SVC (open) and SPA (solid) isolated from mice of 5 weeks, 10 weeks and 20 weeks of age (n=3).

Suppl. Fig. 4 (A) Expression of UCP1 in SPA, SVC, differentiated SPA (D-SPA) and differentiated SVC (D-SVC) (B, C) Protein levels of UCP1 in SPA, D-SPA and brown adipose tissue (BAT) were evaluated by immunoblot analysis. Typical immunoblots (B) and quantified results (C) are shown. Each value shows the average of the relative protein levels (D-SPA as 1) of UCP1 (n=3). (D) Image of SPA treated with or without CL316243 and pioglitazone (E) Expression of Ucp1 mRNA in epididymal SVC (open) and SPA (solid) isolated from mice of 5 weeks, 10 weeks and 20 weeks of age treated with CL316243 and pioglitazone (n=3) (F-H) Results of typical immunoblot analysis. Relative quantified values of UCP1 (G) and PLF (H) in epididymal (open) and inguinal fat (solid) are shown (CL in epididymal fat as 1, n=3)

## Declaration of interest

The authors declare that there is no conflict of interest that could be perceived as prejudicing the impartiality of the research reported.

## Funding

This work was supported in part by the joint research program of the Institute for Molecular and Cellular Regulation, Gunma University.

## Author contribution statement

K Taguchi, K Kajita, Y Kitada, M Fuwa, and T Kajita performed the experiments. K Kajita, T Ishizuka, I Kojima, and H Morita designed the experiments. T Kajita and T Asano analyzed the data. K Taguchi and K Kajita wrote the manuscript.
