# Supplementary material for: Role of small proliferative adipocytes: possible beige cell progenitors
Source: J Endocrinol. 2020 Jan 28;245(1):65–78. doi: 10.1530/JOE-19-0503 (PMC7040459; doi:10.1530/JOE-19-0503)
Supplement: Supplementary Methods [file supplementary_material.pdf]

Supplementary Methods

Preparation of SPA

To obtain an SPA-rich fraction, epididymal or inguinal fat from male C57/BL mice at 10 weeks of age (5-20 mice for each experiment) was resected, and cut into small 1-3 mm pieces. They were digested with 1 mg/mL collagenase (type 1) in Krebs-Ringer phosphate (KRP) buffer supplemented with 1% BSA at 37 C for 20 min with constant shaking, and were passed through silk mesh. Dispersed cells were divided into three fractions by centrifugation. First, the cells were centrifuged at 8 ×g for 1 sec. The floating cells were separated from the lower phase. The floating cell fraction was rinsed three times with KRP buffer, followed by centrifugation at 226 ×g for 3 min. The resultant floating cells and sedimentary cells were regarded as fraction A (mature white adipocytes) and fraction B, respectively. The lower phase of the 8 ×g centrifugation was centrifuged at 226 ×g for 3 min, and sedimentary cells were regarded as fraction C (SVC) (Fig. 1C). Cells in SVC and fraction B were cultured in gelatin-coated 6 well plates with DMEM. Then, we performed the experiments described below to ascertain whether cells in fraction B were SPA. On the other hand, a cell fraction (fraction D) consisting the combination of fraction B and fraction C was obtained by centrifugation of dispersed cells at 226 ×g for 3 min (fraction D). In the present study, SPA were collected from epididymal fat unless otherwise noted. When we compare SPA from epididymal and inguinal fat, the former is termed epididymal SPA and the latter inguinal SPA. To perform immunohistochemical study of beige cells, a specific 3-adrenergic receptor agonist, CL316243 (1 mg/kg/day) was intraperitoneally injected into C57/BL mice for seven days. Animal care and experimental procedures were performed with the approval of the Animal Care Committee of Gifu University Graduate School of Medicine.

Immuno-histochemical and immuno-cytochemical study

For immuno-histochemical studies, epididymal fat obtained from 3 mice was fixed with 10% neutral buffered formalin and embedded in paraffin. The sections were deparaffinized, and antigen retrieval was performed with retrieval buffer and heat at 90 C for 5 min. The sections were blocked with PBS containing 3% BSA for 10 min at room temperature.

For immune-cytochemical studies, 5000 cells/cm^2 obtained from 5-10 mice were plated on gelatin-coated glass bottom plates (Lab-Tek Ⅱ Chamber Slides, Nunc A/S, Roskilde, Denmark). Cells were grown in DMEM until they reached 80% confluence (about 10 days). The cells were washed with PBS, and then fixed with 1% paraformaldehyde for 10 min. The cells were permeabilized with PBS containing 0.5% Triton X for 5-10 min, and blocked with PBS containing 3% BSA for 10 min at room temperature.

The examined tissue sections and cells were incubated with the primary antibodies listed in Suppl. Table 1 for 60 min. They were washed 3 times with PBS, followed by incubation with secondary antibody for 60 min at room temperature, and then counterstained with DAPI. Samples were observed with BZ-II or LSM710 (Keyence, Osaka, Japan; Carl Zeiss, Oberkochen, Germany). The observations were always made in comparison to the negative control generated without primary antibody. The most typical images among 3 independent experiments were shown in the Figures.

To evaluate the proliferative activity of cultured cells, incorporation of 5-ethynyl-2’-deoxyuridine (EdU) was measured. Cultured SPA (confluency 60-80%) were incubated with 10 M EdU for 6 h, and then fixed with 1% paraformaldehyde. Incorporated EdU was detected with a Clik-iT EdU Imaging kit (Invtrogen). The percentage of EdU-positive cells/total nuclei was determined by counting cells manually under a microscope as the proliferative activity.

Quantitative PCR

Samples for real time PCR were obtained from adipose tissue and cultured cells. Cells in fraction B and C obtained from 20 mice were plated in 6 well plate and cultured with DMEM until they reached 90 % confluence.

Quantitative PCR was performed to determine the mRNA levels of various adipocyte-specific genes. PCR amplification was performed as follows: 1 cycle of predenaturation at 95 C for 30 s and 40 cycles of 95 C for 5 s and 60–62 C for 30 s using a Thermal Cycler Dice (Takara). Oligonucleotide primers were shown in Suppl. Table 2. Housekeeping genes were chosen from among the 13 reference genes (Mouse Housekeeping Gene Primer Set, Takara). Since the expression levels of *Gapdh* were stable among various cells, it was designated as the housekeeping gene. Expression levels were quantified using the comparative CT method with housekeeping *Gapdh* and *Acb* levels for normalization.

Immunoblot analysis

Samples for immunoblot were obtained from adipose tissue and cultured cells. Cells in fraction B and C obtained from 10 mice per one sample were plated in flask (25 cm^2) and cultured with DMEM until they reached 90 % of confluence. Cell lysate in one flask was referred to one sample for immunoblot analysis. Three samples were obtained from independent cell cultures.

Equal protein amounts of the cell lysate were subjected to 10% SDS-PAGE, and transferred onto polyvinylidene difluoride membranes. The membranes were blocked with 1% bovine serum albumin and 0.1% Tween-20 in Tris-buffered saline, and incubated with the first antibodies listed in Suppl. Table 1. Protein bands were visualized using secondary antibodies

(Amersham, Buckinghamshire, UK), an Amersham ECL Prime system, and a LAS-4000 Luminescent Image Analyzer (Fujifilm, Tokyo, Japan). Data were quantified using Multi Gauge Ver3.0 software (Fujifilm). The ratio of target genes to actin was shown as the protein expression level.

Microarrays

Total RNA was purified as described above. Microarrays were performed with a Low Input Quick Amp Labeling kit (Agilent Technologies, Santa Clara, CA, USA), SurePrint G3 Mouse 8x60K ver. 2.0 (Agilent Technology), scanned with an Agilent DNA Microarray Scanner (G2505C). Resultant data were analyzed with GeneSpring GX (Ver.12.6).

Flow Cytometry

For Flow cytometry (FCM) analysis, cells in fraction D (SVC+SPA) were suspended in PBS containing 1% FBS. Cells were incubated with or without (negative control) mouse anti-AQP7 antibody (Santa Cruz) and rabbit anti-ADRB3 antibody (Abcam) antibodies listed in Suppl. Table 1 for 30 min. They were washed twice with PBS, followed by incubation with secondary antibody for 30 min at room temperature. FCM was performed with EC800 Cell analyzer (SONY, Tokyo, Japan).
