# Supplementary material for: Role of small proliferative adipocytes: possible beige cell progenitors
Source: J Endocrinol. 2020 Jan 28;245(1):65–78. doi: 10.1530/JOE-19-0503 (PMC7040459; doi:10.1530/JOE-19-0503)
Supplement: Suppl. Table 1 Utilized antibody list. [file supplementary_table_1.pdf]

Suppl. Table 1 Utilized antibody list.

Peptide/protein target	Manufacturer, catalog #, and/or name of individual providing the antibody	Species raised in; monoclonal or polyclonal	Dilution used
Adiponectin	Santa Cruz Biotech (sc-56)	goat polyclonal	50 (IHC)
Leptin	Santa Cruz Biotech (sc-9014)	rabbit polyclonal	50 (IHC), 150 (WB)
Proliferin	Santa Cruz Biotech (sc-271891)	mouse monoclonal	50 (IHC)
Aquaporin 7	Santa Cruz Biotech (sc-376407)	mouse monoclonal	10 (FCM)
GABA receptor gamma 2	Bioss (bs-4112R)	rabbit polyclonal	150 (IHC), 300 (WB)
Synuclein	Novusbio (R-058-100)	rabbit polyclonal	200 (IHC), 500 (WB)
Uncoupling Protein 1	Bioss (bs-1925R)	rabbit polyclonal	100 (IHC), 500 (WB)
Wilmus Tumor 1	Santa Cruz Biotech (sc-393498)	mouse monoclonal	50 (IHC), 200 (WB)
Adrenergic receptor 3	Abcam (ab14713)	rabbit polyclonal	20 (FCM)
beta-actin	Santa Cruz Biotech (sc-47778)	Mouse monoclonal	150 (WB)
