# Supplementary material for: Role of small proliferative adipocytes: possible beige cell progenitors
Source: J Endocrinol. 2020 Jan 28;245(1):65–78. doi: 10.1530/JOE-19-0503 (PMC7040459; doi:10.1530/JOE-19-0503)
Supplement: Suppl. Table 2 Oligonucleotide primers designed based on sequences from the GenBank database. [file supplementary_table_2.pdf]

Suppl. Table 2 Oligonucleotide primers designed based on sequences from the GenBank database.

	Forward	Reverse
Pparg2	GCATCAGGCTTCCACTATGGA	AAGGCACTTCTGAAACCGACA
Cebpa	CAGCTTACAACAGGCCAGGTTTC	GCTGGCGACATACAGTACACACAA
Adipq	AAGGACAAGGCCGTTCTCT	TATGGGTAGTTGCAGTCAGTTGG
Lep	ACCTGTCTACTCATGCCAGCACTC	CTGGTCCTGCAGCCTGTTTG
Aqp7	GAACAGTGAGAAAAAGACCG	CCAGACAATCCAGAGTTCAT
Reten	AAGGCGGAGTTGGCATAGC	ACGAATGTCCCACGAGCC
Adrb3	TGCGCACCTTAGGTCTCATTAT	AAGGCGGAGTTGGCATAGC
Klb	TGTTCTGCTGCGAGCTGTTAC	CCGGACTCACGTACTGTTTTT
Fas	AGATCCTGGAACGAGAACACGAT	CAAGTCCAGGAGTGACACGTCTC
Grm7	CAGTGACAGGCCCAA	TCCATGGGATGGAACAGGTTAGA
Gabrg2	CAGCTTACAACAGGCCAGGTTTC	GGGGCCTTGAAGGAAAACATC
Sncg	CAACACAGTGGCCAACAAGA	GGGGTTCCAAGTCCTCCTT
Plf2	CCATGTGTGCAATGAGGAATG	TGCCGGCTAATTCAAATGTG
Gapdh	GGCATTGTGGAAGGGCTCAT	GACACATTGGGGGTAGGAACA
Pgc1a	AAACTTGCTAGCGGTCCTCA	TGTTGACAAATGCTCTTC
Ucp1	GCCTTCAGATCCAAGGTGAA	TAAGCCGGCTGAGATCTTGT
Ppara	CCATGTGTGCAATGAGGAATG	TGCCGGCTAATTCAAATGTG
Dlk1	TCTGCGAGGCTGACAATGTCTGC	CCTTGTGCTGGCAGTCCTTTCC
Pdgfra	ACACTTTGACCGTCCCCAAG	AAGGTGGGCTCAATCTCGAC
Wt1	TTGGTCCGCCATCACAACAT	ATGCAGTCAATCAGGTGTGCT
Plin1	CTGTCTGAGACTGAGGTGGC	TCAGGGAGGTCTCCATCCAG
Plin4	TCCTGCCCCCTCATCTAAAG	TTAGTTCCGACAAGGGCCGA
Pdgfrb	CTACAATGCCATCAAGAGGG	AACTCCTCATCTACCTGCTG
Myf5	CCACCTCCAACTGCTCTGA	GCTGTCAAAGCTGCTGTTCTT
Zic1	AACCTCAAGATCCACAAAAGGA	CCTCGAACTCGCACTTGAA
Cd137	CTGTGCTTAAGACCGGGACC	CTGGGAGAGAAGCTCACCAC
Tbx1	CGAATGTTCCCCACGTTCCA	GTCTACTCGGCCAGGTGTAG
Ly6c1	ACTGTGCCTGCAACCTTTGTCT	GGCCACAAGAAGAATGAGCAC
Cd9	TGCAGTGCTTGCTATTGGAC	GGCGAATATCACCAAGAGGA
