# Supplementary figures and images for: Role of small proliferative adipocytes: possible beige cell progenitors
Source: J Endocrinol. 2020 Jan 28;245(1):65–78. doi: 10.1530/JOE-19-0503 (PMC7040459; doi:10.1530/JOE-19-0503)

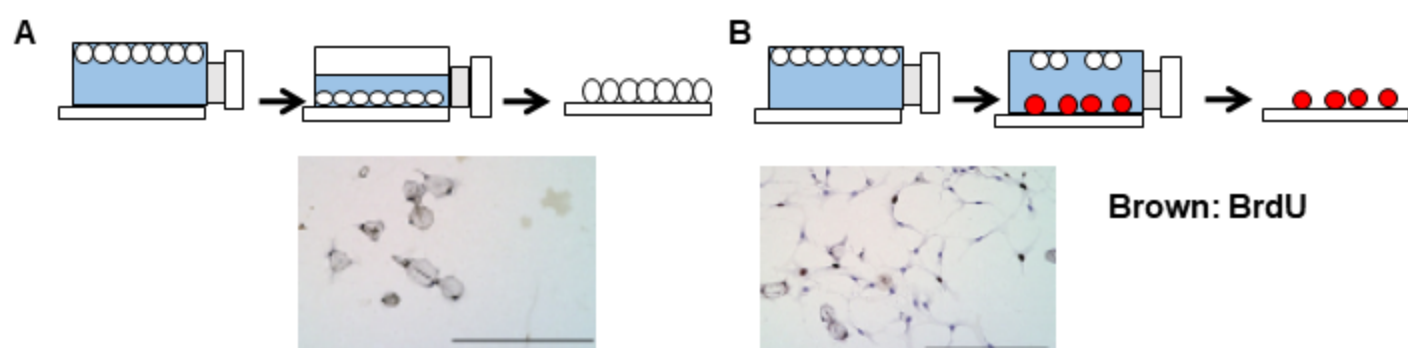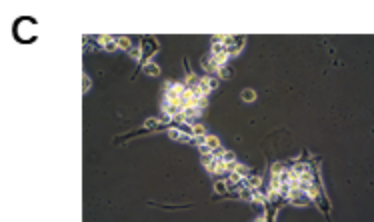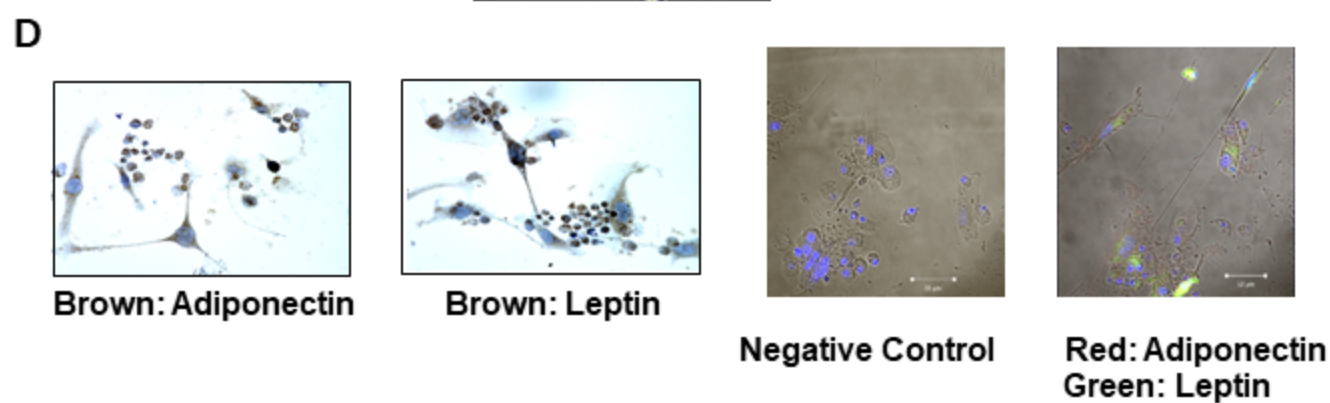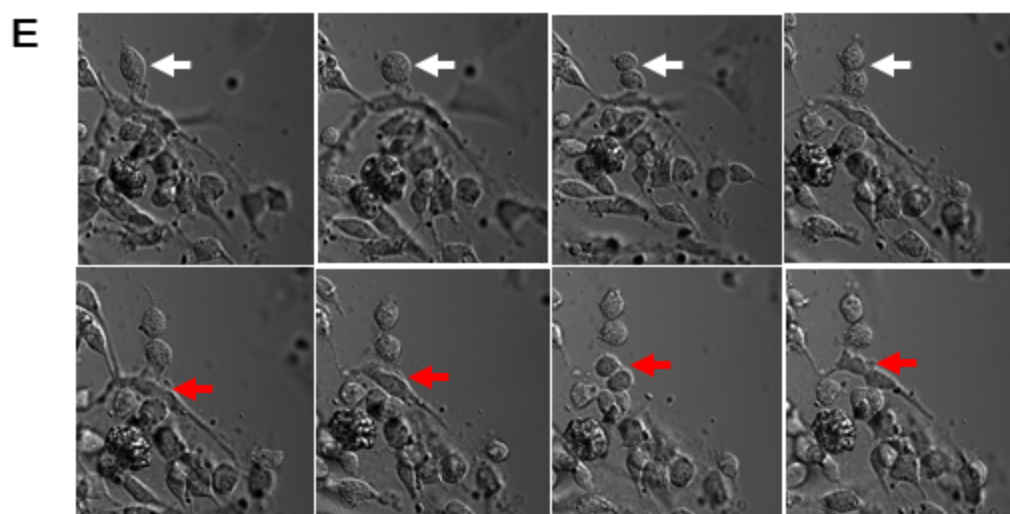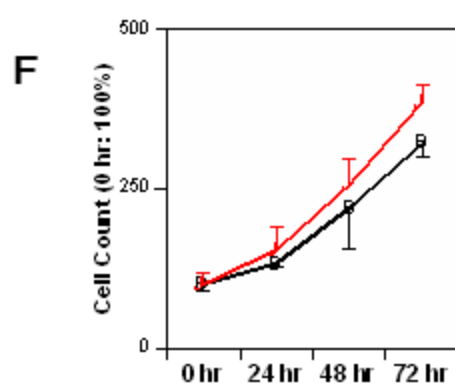

Supplement: Suppl. Fig. 1 (A) Ceiling culture (upper panel) and cultured cells (lower panel). Floating cells isolated from five C57/BL mice were applied. Cultured cells were treated with BrdU (10 μM) for 12 h. Incorporated BrdU was visualized using anti-BrdU antibody. (B) Reverse ceiling culture (upper panel) a [file supplementary_figure_1.pdf]

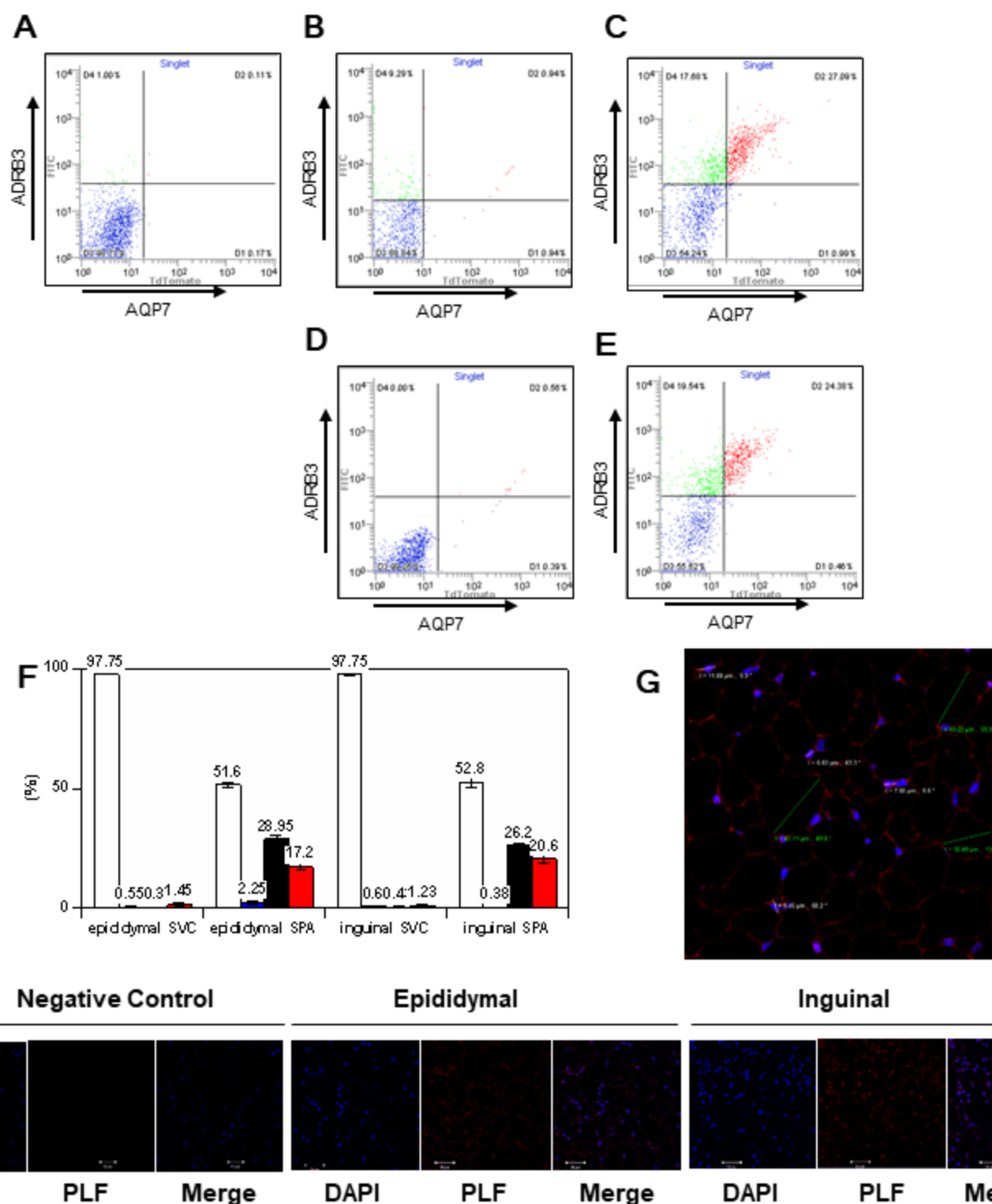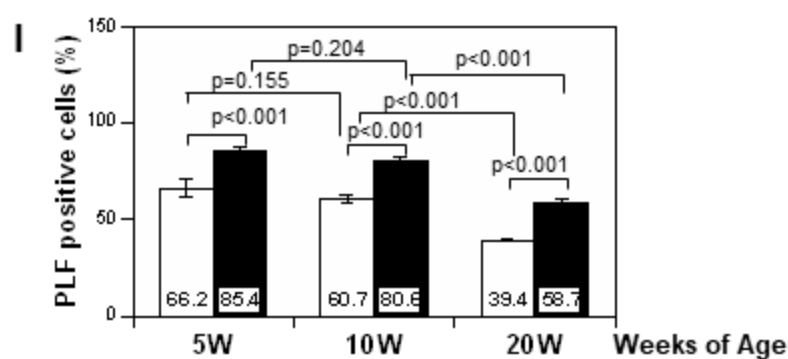

Supplement: Suppl. Fig. 2 (A-E) Flow cytogram of AQP7+; ADRB3+ cells in negative control (A), epididymal SVC (B), epididymal SPA (C), inguinal SVC (D) and inguinal SPA (E). (F) Percentage of AQP7-; ADRB3- cells (white), AQP7+; ADRB3- cells (blue), AQP7+; ADRB3+ cells (black) and AQP7-; ADRB3+ cells (red) were s [file supplementary_figure_2.pdf]

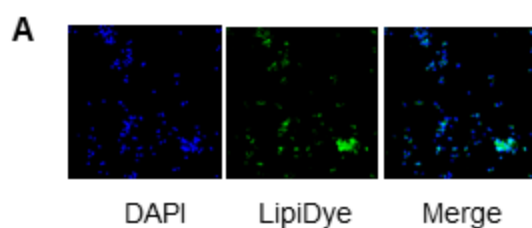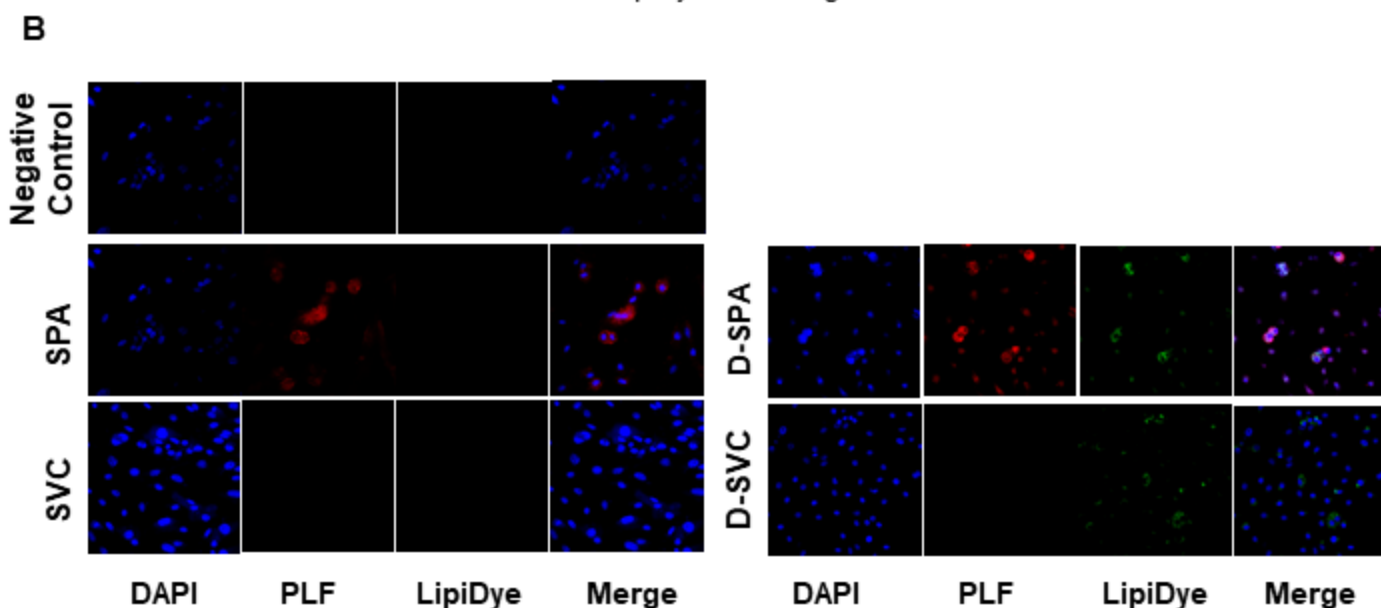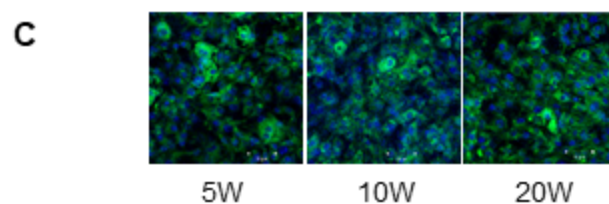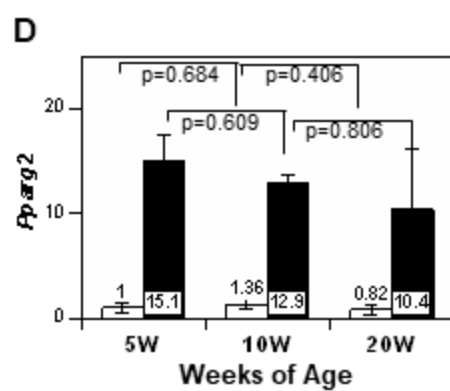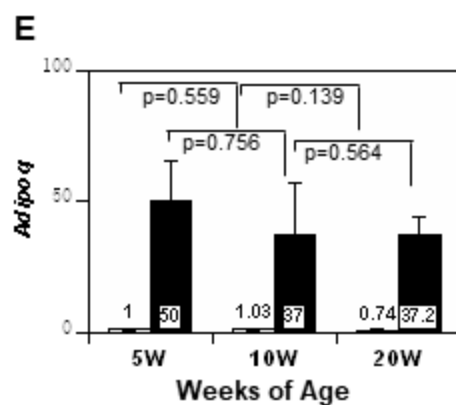

Supplement: Suppl. Fig. 3 (A) Typical image displaying clustered round cells that were easily differentiated into lipid-laden cells. (B) Expression of PLF in SPA, SVC, differentiated SPA (D-SPA) and differentiated SVC (D-SVC) (C) Lipid-laden cells in adipogenic differentiated epididymal SPA isolated from mice o [file supplementary_figure_3.pdf]

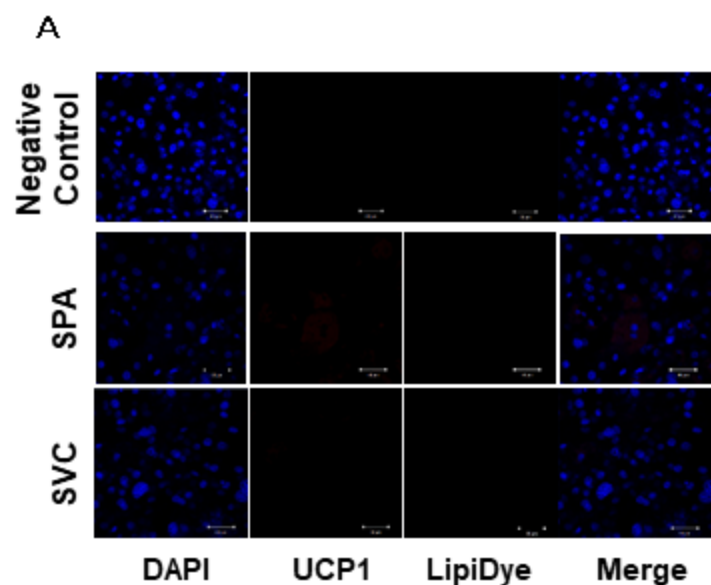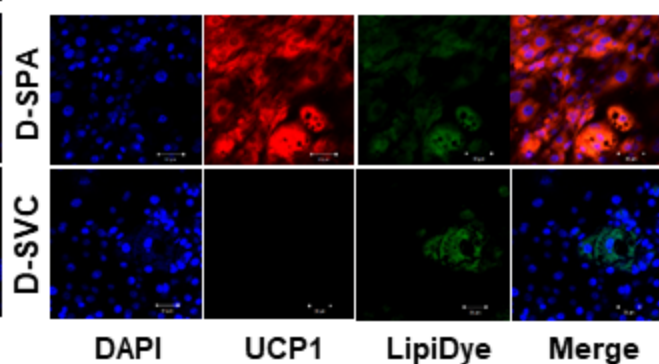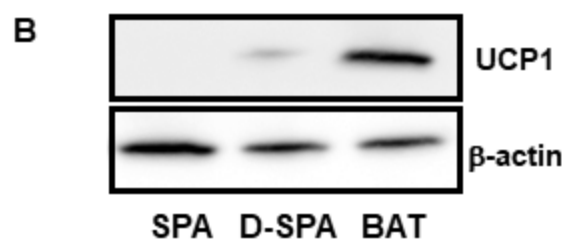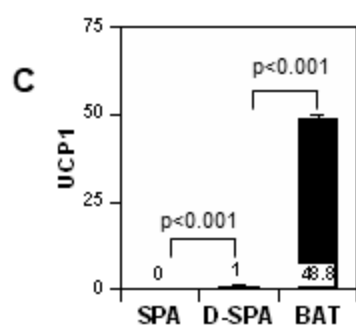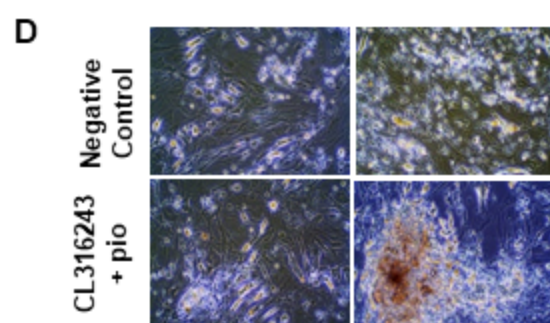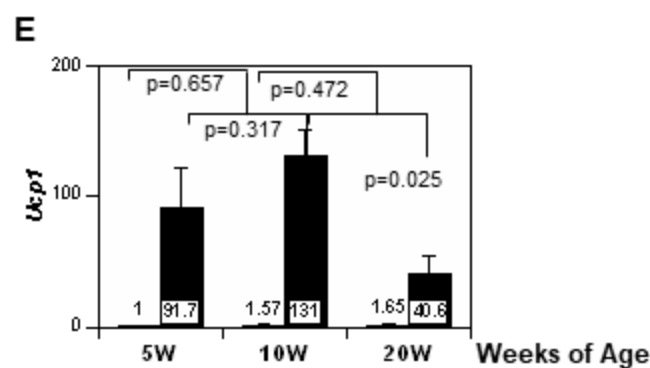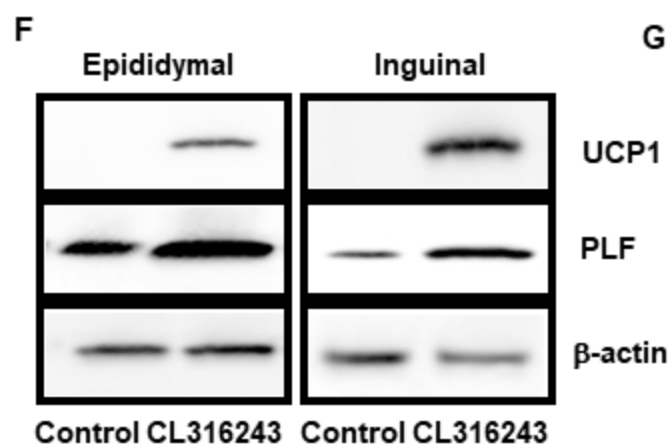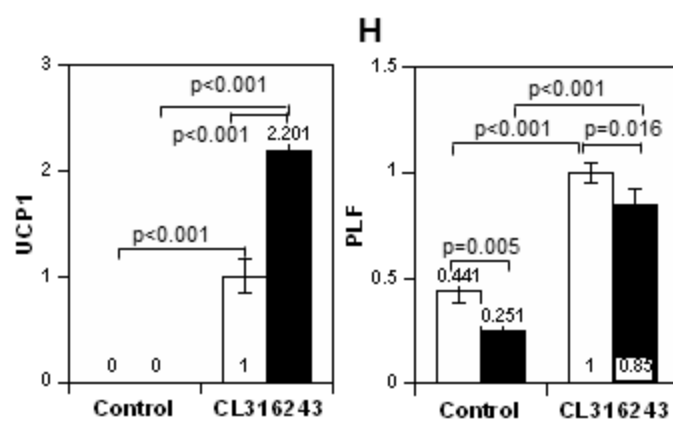

Supplement: Suppl. Fig. 4 (A) Expression of UCP1 in SPA, SVC, differentiated SPA (D-SPA) and differentiated SVC (D-SVC) (B, C) Protein levels of UCP1 in SPA, D-SPA and brown adipose tissue (BAT) were evaluated by immunoblot analysis. Typical immunoblots (B) and quantified results (C) are shown. Each value shows [file supplementary_figure_4.pdf]
